# The HR revolution: Redefining performance paradigms in Pakistan’s pharma landscape through moderating role of innovative climate

**DOI:** 10.1371/journal.pone.0301777

**Published:** 2024-05-31

**Authors:** Abdul Waheed, Salma Waheed, Shahbaz Hussain, Abdul Majeed

**Affiliations:** 1 School of Management, Jiangsu University, Zhenjiang, China; 2 School of Psychology, Shaanxi Normal University, Yanta District, Xi’an, China; 3 School of Management Science, University of Okara, Okara, Pakistan; 4 School of Management Science, University of Lahore, Lahore, Pakistan; University of Central Punjab, PAKISTAN

## Abstract

The main objective of this research is to examine whether the implementation of new human resource management practices (NHRM) can enhance innovative performance (IP) by fostering innovation capability (NC). Additionally, it examines the moderating influence of an innovative climate (IC) and its associated attributes on the association with new human resource management and innovation capability, a factor that has been relatively overlooked in prior research. The study’s data was obtained from 398 employees in the pharmaceutical sector of Pakistan, and hierarchical regression analysis was utilized to analyze the data. The results from the mediating and moderating analyses underscore the significance of NHRM practices, innovation capability, and an innovative climate, along with its specific attributes, in promoting innovative performance through factors such as operational efficiency, suitable practices, and employees’ willingness to participate in organizational endeavors. Furthermore, the moderated mediation analysis findings reveal that the influence of innovation capability as a mediator is strengthened when the workplace climate is more conducive to innovation. These findings have implications for both theoretical understanding and practical application, particularly in similar developing countries. The study offers insights that can be generalized to developing nations with comparable economic and social structures.

## Introduction

The increasing trend of globalization and the ever-changing nature of business environments are compelling companies worldwide to prioritize innovation to address the continuous development of challenges and possibilities. The intellectual capital of a corporation is closely intertwined with its ability for innovation, considering it from the organization’s perspective [1]. In the contemporary corporate environment, which is marked by persistent rivalry and constant changes, the capacity to foster innovation has emerged as a critical determinant of achieving success as an organization and sustaining long-term significance [2]. In this context, a significant responsibility for managers lies in formulating new human resource management (NHRM) initiatives aimed at cultivating and enhancing the intellectual capital assets of their organizations. This, in turn, contributes to enhancing innovation capabilities and overall organizational performance [3]. Human resource management (HRM) practices play a crucial part as the primary mechanisms through which firms exert influence and mold the competencies and behaviors of their personnel to accomplish organizational objectives [3,4]. While several prior types of research have concentrated on the relationship between HRM practices and several outcomes, including flexibility, efficiency, or economic performance [3,5], we need to expand our understanding to include measures of innovation. This variable has significant importance for several organizations that are endeavoring to build and maintain a competitive edge [5,6]. As a result, a significant concern for these companies revolves around devising and implementing coherent sets of practices that can enhance their innovation capabilities.

Innovative performance stands as a pivotal concept in the area of human resource management (HRM) and organizational governance, serving as a means for firms to attain various competitive objectives and confront challenges [7–9]. Existing literature underscores the imperative of enhancing innovative performance as a foundational factor for companies to gain strategic advantages and ensure sustainable development [8]. Nevertheless, to companies operating in unfamiliar and growing markets, transitioning from imitators to genuine inventors poses a formidable challenge. This challenge stems from the prevalence of medium to small firms that often grapple with limited capital and resources for innovation [10,11]. Consequently, researchers and practitioners have dedicated significant efforts to identifying the underlying determinants and novel solutions to bolster innovative performance for these firms [11]. The current academic research on the relationship between new human resource management (NHRM) practices and firm innovative performance is limited in the field of HRM. However, a few studies by Foss and Laursen [12] and Sung and Choi [13] suggest that NHRM practices may have a positive impact on firm innovative performance.

Nonetheless, as stated by Kianto, Ritala [14], there are several unexplored factors that hold the potential to influence the aforementioned relationship. Recent research conducted by Cao, Le [8] and Iqbal [15] have examined how knowledge sharing acts as a mediator between NHRM practices and the potential of organizations to innovate. Additionally, Capelleras, Domi [16] delved into the mediation of firm performance by innovativeness through skill-enhancing HRM practices. Notably, the existing body of literature has largely overlooked the part played by innovation capability in stimulating a firm innovative performance. This underscores the necessity of examining the mechanisms that underlie the connection with NHRM practices and firm innovative performance. However, research addressing the linkage among NHRM and innovation capability remains relatively scarce. The significance of nurturing innovation capability has garnered growing attention, especially in situations characterized by dynamism and uncertainty [1]. Innovation capability, defined as an organization’s capacity to generate and implement novel ideas, technologies, processes, and business models, has gained significant attention in academic discussions. This discussion revolves around strategies to improve competitiveness and achieve sustainable success over the long term [5,17]. This study delves into this critical link and aims to reveal how newly implemented HRM strategies influence innovation in pharmaceutical companies. [18]. The literature asserts that organizations must embrace innovation activities by leveraging NHRM practices to enhance employee engagement in the pursuit of new knowledge and originality [3,12]. Furthermore, prior research has suggested that innovation capability can serve as a catalyst for improving the innovative climate within an organization [11]. To effect change in the organizational system, which necessitates both operational and administrative adjustments, optimizing organizational outcomes is imperative. Nevertheless, the aspect of innovation within pharmaceutical organizations has received limited scrutiny and discussion [2,5]. More recently, scholars have displayed a keen interest in understanding the impact of innovative climates, along with their five key attributes: working environment support (WES), leader support (LS), resources supply (RS), learning and growth (LG), and knowledge and skills (KS), within pharmaceutical organizations. This interest stems from the recognition that an innovation climate promotes novel work tactics, impacts risk-taking behavior, facilitates the adoption of new technologies, and cultivates a stimulating work environment within an organization [18].

The adoption of technological processes holds significant importance and serves as the foundation for making challenging decisions. Consequently, a thorough investigation of technological information adoption is advisable [9]. This examination provides insights into the robustness of the theoretical framework’s conceptualization along with the interplay of particular factors [18]. While rational frameworks tend to foster reluctance towards change, eco-friendly factors play a pivotal role in influencing technology selection decisions and the level of satisfaction attained (30, 31). This emphasizes predictability in the process over the creative aspects of individuals, aligning with the flexible focus of contemporary theorists [6]. Several theoretical frameworks, including the T-O-E and RBV theory, the knowledge-based viewpoint, and the human capital theory, have been employed in previous research. to establish the connections between NHRM, innovative performance (IP), and innovation capability in pharmaceutical firms [19,20]. Furthermore, scholars have posited that the Technology Organization Environment (T-O-E) and Resource Based Theory (RBV) paradigm, frequently employed in the context of creative performance, substantiates an organization’s holistic aptitude, efficacy, operational efficiency, and methodologies [21]. The T-O-E and RBV theories also prove valuable worth when applied to NHRM practices, offering sustainable competitive advantages [22]. NHRM practices can be characterized as distinct, valuable, unique, and noteworthy due to their capacity to enable the sustainable application of the T-O-E and RBV theory within organizations [23,24]. This holds significant relevance within the realm of pharmaceutical organizations, where the attainment of a competitive advantage is crucial. This study empirically employs the integrated TOE and RBV theories to analyze the connection between innovation capability and innovative performance [25,26]. Moreover, the link across NHRM and its direct and indirect effects on innovative performance via innovation capability is investigated.

Although there is a well-established connection between NHRM and its significant impact on innovative performance, particularly in the realm of pharmaceutical companies, this favorable impact raises the inquiry as to how NHRM brings about these positive effects [21]. Organizations should strive to provide a nurturing atmosphere for their people that promotes positive behaviors and transforms the overall organizational climate to allow continued discovery. One way to reshape the organizational climate is through the encouragement of innovative behavior, resulting in what is termed an innovative climate, encompassing attributes like working environment support (WES), leader support (LS), resources supply (RS), learning and growth (LG), and knowledge and skills (KS).

The overall objective of this study is to contribute to the existing body of knowledge by examining a moderated-mediation model. This research aims to make a scholarly contribution to the current body of literature and management practices. NHRM practices play an effective role in fostering these climates within pharmaceutical firms and contribute to the creation of a significant working environment. Nevertheless, a dearth of literature delves into these topics, particularly within the context of the pharmaceutical sector. Researchers have underscored the need for future research to explore more intricate relationships between NHRM practices, the innovative climate, and its associated attributes [8,16]. Furthermore, the discussion around innovation capability and NHRM has been relatively limited [2,27], making it a pressing issue that has been termed a research gap requiring attention. The research promotes knowledge transfer and collaboration between academia and industry by converting academic research findings into valuable insights for real-world applications. The research disseminates findings through academic publications, industry reports, and professional conferences, fostering dialogue and collaboration between researchers and practitioners promoting mutual learning and innovation. As a result, this present investigation seeks to empirically analyze the mediation effect of innovation capability between NHRM and innovative performance while also considering the moderating impact of the innovative climate and its attributes (WES, LS, RS, LG, and KS) on the association between NHRM and innovation performance in the pharmaceutical sector in Pakistan.

### Literature review and development of hypothesis

#### New human resource management, innovation capability, and innovative performance

The Resource Based (RBV) theory was initially introduced by Barney [28] ] and the T-O-E theory by Tornatzky, Fleischer [29] more than two decades ago. Since its inception, researchers have dedicated their efforts to understanding its impact on organizational outcomes [30,31]. From a strategic human resource management (SHRM) perspective, the implementation of new human resource management (NHRM) practices is seen as a critical element in ensuring growth, success, skill enhancement, behavioral improvement, and competence development to achieve innovation capability [24,32]. Researchers also assert that NHRM practices place emphasis on many factors, such as digital recruitment and selection, unique remuneration techniques, progressive training, and innovative performance. These factors are believed to have a substantial impact on fostering employees’ creativity inside firms [33]. Such innovative behavior within the context of NHRM practices stimulates motivation for innovation capability, which, in turn, positively influences innovative performance [32,33]. The implementation of innovative ideas by employees can significantly boost their confidence, ultimately leading to enhanced innovation capability. Motivating employees profoundly impacts innovative performance through HRM practices [34]. Researchers have also presented empirical support for HRM’s influence on employee performance within the context of organizations, emphasizing the role of motivation in improving employees’ abilities and performance levels [24,34]. Similarly, NHRM practices hold the possibility to elevate employee motivation and improve innovative performance by promoting and reinforcing these practices[35]. It’s worth noting that innovation capability is a crucial factor in maintaining a sustainable competitive advantage. Hurtado-Palomino, De la Gala-Velásquez [11] have highlighted those enterprises can secure a lasting competitive edge by continually enhancing their independent innovation capabilities. Collectively, these prior studies have established a robust theoretical foundation for the expectation that innovation capability contributes to enhanced innovative performance within firms.

#### New human resource management, innovative climate, and innovation capability

Organizations have been compelled to adapt to environmental changes, and highly knowledgeable ones have turned to innovative systems to ensure their business survival. Innovation capability revitalizes employees, enhancing their motivation, knowledge, skills, and performance. Researchers have regarded HRM practices as a dynamic element of innovation capability, as prior literature has focused on the presence of innovation within HRM [2], the emergence of innovation, innovation process systems [15], and the outcomes of firm performance [30]. Therefore, it is evident that HRM practices have a significant influence on innovation capability. In recent years, HRM practices have evolved to include New HRM practices (NHRM) and their relationship with innovation [12,33]. Additional investigation is necessary to clarify the correlation between NHRM practices and innovation capability since in-depth studies may better comprehend the processes that connect NHRM practices and innovation capability.

Within the framework of NHRM practices and their connection with innovation capability, the innovative climate can play a vital role as a moderator. Innovative climate dimensions have been employed in various domains of innovation research, including national innovation [18], cross-cultural customer innovation, and adoption of innovation [16,36]. Initially, the concept of organizational climate was seen as a rather generic construct encompassing various dimensions influencing employee performance [37]. However, more recently, organizational HRM has started to focus on the working climate in which employees operate. It is essential for employees’ schemas in perception and interpretation to be positive [38], as organizations highly value positive employee behavior. Organizations also foster a supportive environment that is reinforced through incentives, which contributes to a favorable climate [39]. Such a positive climate provides the necessary support for innovation. Additionally, researchers have emphasized the significance of the climate as a structural element that connects organizational practices with employee behavior [38].

#### New human resource management and innovative performance

Innovation outcomes frequently arise due to the activities involved in creating, choosing, and developing creative ideas, eventually culminating in the provision of novel products or services to various markets [40]. This entails utilizing information, competences, and human resources to fulfill the organizational requirements for innovation [8]. In the realm of business, innovative performance refers to the degree to which companies effectively bring out original innovations to the market. This encompasses several aspects, including the frequency at which they offer new goods, process systems, or equipment [11]. In its essence, innovative performance encompasses the generation and implementation of original components that enhance the long-term efficiency of a company and establish a distinct competitive edge [15]. According to the HRM literature, innovative performance is facilitated by HRM through an integration of HRM practices and the appropriate strategic HRM perspectives [9,41]. Knowledge-human resource management (KHRM) practices are considered crucial decisions in the current knowledge-intensive era. These practices empower organizations to gain access to exceptional and valuable knowledge, which subsequently impacts their capacity for innovation and overall performance [42]. Academics acknowledge that NHRM practices may be implemented as collections of management tasks intended to draw in, keep, and inspire workers to learn, share, produce, and use knowledge [24,43]. These bundles include NHRM practices, including incentive systems, teamwork, digital recruitment and selection, education and training, and employee participation. The body of research generally sees NHRM practices as a management strategy that centers on drawing in, keeping, and inspiring workers to learn, share, apply, and create knowledge resources to maintain competitive advantages and realize companies’ long-term growth [22,24].

Numerous studies within the expanding HRM literature have demonstrated the essential and beneficial influences of HRM practices on innovation. Diaz-Fernandez, Pasamar-Reyes [44] stated that HRM practices serve as antecedents of invention, emphasizing that firms should allocate resources towards HRM practices to effectively leverage for stimulating invention. They drew on the Spanish survey of industrial strategic behavior and longitudinal analysis spanning from 2001 to 2008. HRM practices according to Aman, Noreen [45], are intended to create an atmosphere that supports the growth of employees’ abilities and competences for innovation. Their results demonstrated how HRM policies greatly improve workers’ capacity for innovation at banks in Vehari, Pakistan. Similarly, Bondarouk, Marsman [33] suggested that new HRM (NHRM) methods help staff members gain knowledge and intellectual capital, which can eventually lead to better performance in terms of innovation. Kutieshat and Farmanesh [24] NHRM practices are a collection of thoughtfully chosen HR practices intended to improve organizational knowledge and human capital to co-create relevant experiences and boost creative performance. Based on the aforementioned arguments, this study formulates the following hypotheses.

H1: NHRM practices have a favorable connection with innovative performance.

#### Innovation capability as a mediator

Innovation capability encompasses an organization’s overall capacity and resources for consistently enhancing its ability to explore and exploit opportunities, aiming to develop new products that satisfy the market’s needs [3,46]. This study delves into the intermediary effect of innovation capability in the connection involving NHRM practices and organizational innovative performance, with a focus on the T-O-E and RBV theory. This study incorporates existing literature to develop the theoretical foundations of the mediator. The literature emphasizes an indirect link that is mediated by the innovation capability (NC). This capability assists as a bridge between NHRM practices and innovative performance. However, previous research has predominantly focused on examining the impact of innovation on the performance of exports and globalization. This research adopts the T-O-E by Tornatzky, Fleischer [29] and the RBV theory introduced by Barney [28] to substantiate the role of innovation capability, drawing from the organizational factors outlined in the RBV literature. A company’s size, competencies, resources, communication channels, organizational structure, and other traits are all considered organizational variables [21,25]. While limited studies have explored the mediation role of innovation in this context [16,47].

Iddris, Mensah [2] have identified that the existence of supportive HRM practices and innovation capabilities acts as a mediator in the immediate effect of a customer-oriented approach on financial performance. This emphasizes the crucial significance of SHRM strategies in stimulating employee motivation towards engaging in innovation, thereby leading to corporate achievement. Similarly, Yusr [48] pointed out that businesses dedicated to improving their innovation capability can effectively execute dynamic and innovative procedures. Within the realm of innovative product performance, there exists a significant intermediary function that connects organizational learning capability with export competitiveness. It has emphasized the role of pharmaceutical flexibility as a mediator in conjunction with organizational capacity to enhance innovative performance and develop innovation skills. Building on the insights from previous literature, our study puts forth the proposition that innovation capability plays a crucial role in mediating the relationship between the implementation of new human resource management (NHRM) practices and the subsequent level of innovative performance. Consequently, we formulate the hypothesis below:

H2: The association within NHRM practices and innovative performance is mediated by innovation capability.

#### Moderating effect of innovative climate

Prior research has underscored the importance of paying consideration to the limitations and contextual factors associated to new HRM (NHRM) practices and has specifically focused on pharmaceutical organizations [32,49]. The introduction of the idea of an innovation climate serves as an intermediate within NHRM practices and innovation capabilities, with the purpose of addressing the moderating effects. The innovative climate operates as a dynamic process inside an organization, prospering in a creative setting where employees’ active work behaviors are in harmony with the prevailing working conditions [49]. While previous research has explored the potential moderating effects of the organizational climate recent investigations have shifted their focus to the broader influence of global organizational climate [45,50]. Nevertheless, some scholars have honed in on specific climates, particularly the "innovation" climate [18] An innovative climate is a presence within organizations that can facilitate the impact of NHRM practices on innovation capability. Amabile, Conti [51] have delineated the innovative climate using eight dimensions, referred to as KEYS: "knowledge and skills, leader support, work environment support, freedom, resources supply, learning and growth, workload pressure, and organizational impediments." These dimensions provide a valuable framework for identifying the key factors that influence innovation capability in the workplace. Building on this foundation, as cited by West, and Hirst [52], the innovative climate has been distilled into four key dimensional scales, including leader support, knowledge and skills, learning and growth, and support for innovation.

For a comprehensive understanding of the innovative climate, our focus centers on five dimensions: work environment support, leader support, resource supply, learning and growth, and knowledge and skills. These dimensions and their associated concepts draw from a diverse body of supporting literature, as detailed in Table 1. The debate surrounding the direct impact of NHRM practices on innovative performance has prompted inquiries into the organizational environments that influence technology utilization and facilitate the achievement of business objectives. The relevant literature underscores the significant role of an innovative climate in fostering a positive impact on technology usage [39]. Prior research has established a clear connection between employees’ innovative behavior and the presence of an innovative climate, along with its five key dimensions (WES, LS, RS, LG, and KS) [53]. Employees who exhibit such innovative behavior actively contribute to the development of a creative atmosphere in the workplace [39].

**Table 1:** Dimensions of innovative climate.

Dimension	Concepts	Literature
Working Environment Support	The extent to assessments of aspects of organizational work environment perceptions that encourage and support new ideas and innovative approaches and is oriented towards change.	[1–10]
Leader Support	The extent to which employees experience support and understanding from their Leadership.	[1,2,4,6,9–11]
Resources Supply	The extent to which employees experience support with the required resources to implement their innovativeness.	[1,2,4–6,8–10]
Learning and Growth	The extent to which employees timely experience rewards and promotions motivates them towards a job and loyalty to the organization.	[1,2,4,6,8–11]
Knowledge and Skills	The extent to which employees learn a new skill or undergoing training is the sense of achievement.	[1,2,4,6,8–11]

Scholars have advocated for an improved approach that centers on "criterion-oriented climates" [54], built on the foundation of a "climate for innovation." This kind of climate fosters creativity in the workplace [55] and actively supports innovation. Shalley and Gilson [56] have shed light on how an innovative climate describes how innovation-driven organizations employ the proper methods and tools and provide the necessary resources to motivate employees to innovate. New HRM (NHRM) practices play a pivotal role in encouraging employee performance at their best, reinforcing their work, and enhancing the innovative climate across its five dimensions: WES, LS, RS, LG, and KS. These dimensions of the innovative climate, in conjunction with NHRM practices such as digital recruitment & selection, progressive and training, reward systems, teamwork, and employee participation, create a supportive environment where innovation capability can thrive [57]. The hypotheses derived from this discussion are as follows.

H3a: Working environment support has a moderating role among NHRM practices and innovation capability.

H3b: Leader support has a moderating role among NHRM practices and innovation capability.

H3c: Resources supply has a moderating role among NHRM practices and innovation capability.

H3d: Learning and growth have a moderating role among NHRM practices and innovation capability.

H3e: Knowledge and skills have a moderating role among NHRM practices and innovation capability.

Determining the factors influencing the utilization of NHRM-related assets and resources is of utmost importance, and it has significantly transformed the nature of collaboration between organizations and their networks. The specifics of this collaboration depend on the context, and it relies on organizations’ trust in combining their assets, resources, and services. Such collaboration has the potential to yield more competitive and innovative outcomes [49,58]. These innovative outcomes can, in turn, serve as a catalyst for enhanced competitive advantage from NHRM practices, ultimately contributing to innovative performance and success. From this perspective, the existing literature highlights the concept of an innovative climate, which has been shown to positively influence innovative performance [38]. Following the T-O-E and RBV theory, the innovative climate plays a critical role in conveying the workplace environment to employees. This involves recognizing innovative performance as a valued behavior within the organization, and employees, in turn, can effectively reciprocate the organization through their innovative contributions [54]. The communication effectively conveys to employees that creative achievements are highly respected. As a result, this innovative climate will likely amplify the positive influence of NHRM practices on innovative performance by fostering a creative and adventurous atmosphere. For instance, if senior management exhibits supportive behavior that enhances creative thinking, and individuals perceive an innovative climate where they can take initiatives without the fear of retaliation or ridicule and have granted adequate, it can further stimulate innovation within the organization [43,55].

An intermediary model that clarifies the impact of innovative climate as moderator is further presented based on Hypotheses 2 and 3. This research suggests that a conducive innovative climate is more favorable; NHRM practices exert a more substantial effect on innovation capability, leading to a pronounced enhancement in innovative performance. Consequently, the impact of NHRM practices on innovative performance, mediated by innovation capability, will be less pronounced. In summary, the discussions above culminate in the formulation of a final hypothesis: innovative climate with its attributes performs the moderating role. This research posits that when the innovative climate is more conducive, NHRM practices will have a more substantial impact on innovation capability, resulting in a significant improvement in innovative performance, while the effect of NHRM practices on innovative performance, mediated by innovation capability, will be attenuated.

H4a: Working environment support moderates the degree of strength of the mediation impact within NHRM practices and innovative performance via innovation capability.

H4b: Leader support moderates the degree of strength of the mediation impact within NHRM practices and innovative performance via innovation capability.

H4c: Resources supply moderates the degree of strength of the mediation impact within NHRM practices and innovative performance via innovation capability.

H4d: Learning and growth moderate the degree of strength of the mediation impact within NHRM practices and innovative performance via innovation capability.

H4e: Knowledge and skills moderate the degree of strength of the mediation impact within NHRM practices and innovative performance via innovation capability.

**Fig 1:**
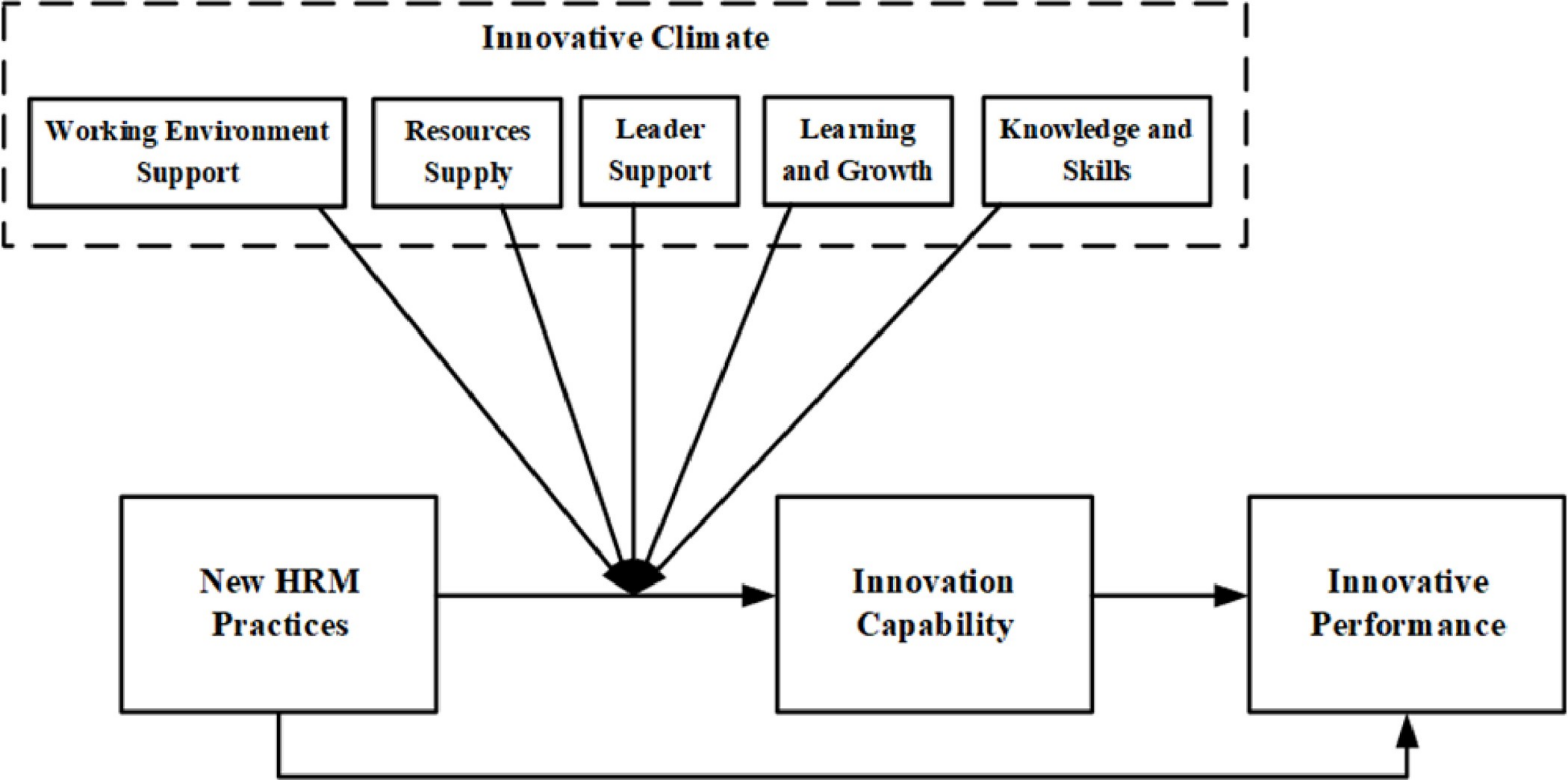
Conceptual model of study.

Based on the theoretical analysis of the literature discussed above, this study presents a moderated mediator model to explain how NHRM practices support innovative performance in the Pakistani pharmaceutical sector. Within this particular paradigm, the concept of innovation capability assumes the role of a mediator between the practices associated with the NHRM and the resulting innovative performance. The impact of innovation capability is further regulated by the aspects of the innovative climate (WES, LS, RS, LG, and KS). The model utilized in the investigation is depicted in Fig 1.

### Methodology

At the juncture of Pakistan’s independence, the pharmaceutical sector was non-existent. In Pakistan, however, more than 400 pharmaceutical companies, including domestic and international enterprises, are operational. These national firms contribute significantly to addressing various health-related challenges. Despite being in a developing stage, the pharmaceutical industry has experienced notable growth. The Pakistan Pharmaceutical Manufacturers Association (PPMA), established in 1961, serves as a representative body for this industry, encompassing more than 400 firms across Pakistan [49,59]. Over the past five years, the Pakistani economy has demonstrated an annual growth rate of around 6.6%, with the pharmaceutical sector experiencing approximately 11% growth (IMS Health Pakistan, 2015) [50]. Recognized for producing high-value-added life-saving products, pharmaceutical industries in Pakistan are crucial knowledge-intensive organizations; the analysis in this study focuses on individual employees as the primary unit of analysis from 47 companies.

The research has been granted ethical clearance by the Central Academic Review Board (CARB) of the School of Management Sciences at the University of Okara. Informed written consent was obtained from all study participants, who claimed their understanding of the study’s purposes and they would like to participate in the study voluntarily. The respondents were assured that their identities and responses were kept confidential which would be maintained in the best interests of academic integrity and every possible manner. Additionally, they were informed of the importance of the study, its possible implications, their role as participants, and that no compensation would be offered. Respondents were assured that they could stop responding and withdraw from the interview at any time. The data collected will be used only to suggest mechanisms for abating the impostor’s feelings through academic outcomes. We contacted staff members at different organizational levels, including upper, middle, and front-level staff. These individuals possess comprehensive knowledge of organizational practices and policies. To ensure the questionnaire’s validity, informal interviews were conducted with department managers having middle or upper positions before distributing the questionnaire to the organizations. During these interviews, officials were asked to identify ambiguous, imprecise, or unclear phrases and provide feedback to enhance the questionnaire’s clarity and relevancy. The questionnaire was refined in light of the department leaders’ vital feedback. Due to security and confidentiality concerns, this study could not secure permission to use the local server network of the organizations for survey responses. Consequently, a systematic survey was formulated utilizing Google Forms (https://forms.gle/AGBg2vCSjm28q9EM7), and the WhatsApp platform was utilized to disseminate surveys to gather data. Voluntary individuals were consulted to obtain valuable responses. The decision to collect data online was based on its effectiveness, error-free nature, and practicality for participants and investigators. However, employees were encouraged to complete the questionnaire during their work hours, taking into account the responsibilities of their positions. In addition, the data collection period is from February 2023 to August 2023.

Considering the context of the study, it utilized a convenience sampling technique along with a purposive sampling technique. Consequently, in adherence to a consensus among the authors, a co-author endeavored to gather data personally through visits to diverse offices and locations. Furthermore, the human resources departments of the chosen organizations extended assistance in establishing correspondence with the participants via WhatsApp platform. The questionnaires were distributed over a period of more than six months to a total of 740 employees, resulting in 398 valid responses to the distributed questionnaires. The questionnaires were divided into two parts. The first part includes survey questions related to demographic items., and the second part includes questions related to this study’s key variable, which are presented in Table 2.

**Table 2:** Survey participants description.

Survey Items	No. of Participants	(%)
Gender		
Male (M)	218	55%
Female (F)	180	45%
Marital Status		
Married	170	43%
Single	228	57%
Age Group		
20–30	90	23%
31–40	115	29%
41–50	136	34%
Over 50 years	57	14%
Education		
Technical Degree	80	20%
Bachelor	117	29.4%
Post Graduate	145	36.4%
MPHIL	51	13%
PhD	5	1.2%
Managerial Level		
Coordinator	102	26%
Supervisor	141	35%
Senior manager	155	39%
Experience in Organization		
Less than 1 year	65	16%
1–5 years	112	28%
6–10 years	95	24%
11–15years	70	18%
More than 15 Years	56	14%

#### Measures

As shown in Table 3, the measurement instruments for all variables were modified from prior research to fit the context of the present investigation. The evaluation of 16 items was conducted to assess the independent variable of new HRM practices. Participants’ responses were collected using a five-point Likert scale from 1 (strongly disagree) to 5 (strongly agree), which was designed by Veth, Korzilius [60] and Waheed, Miao [7]. The reliability of these 16 items fell within a range of 0.61 to 0.83.

**Table 3:** Scale dimensions and variables measures of study.

Sr. No	Study Variables	Code	Authors of Instruments	No of Items	Measurement Scale
1	New Human Resource Management Practices	NHRM	[12,13]	16	1 = Strongly Agree, 5 = Strongly disagree
2	Innovation Capability	NC	[14]	6	1 = Strongly Agree, 5 = Strongly disagree
3	Innovative Climate	IC	[1–3]	20	1 = Strongly Agree, 5 = Strongly disagree
4	Innovative performance	IP	[15,16]	6	1 = Strongly Agree, 5 = Strongly disagree

Respondents from the organizations were asked to indicate the extent to which each new HRM practice closely aligned with their organization, with higher scores indicating greater significance and vice versa. The results of confirmatory factor analysis (CFA) are acceptable (χ2 = 12.118; Df = 7; p < 0.05; CFI = 0.991; TLI = 0.86; IFI = 0.994; RMSEA = 0.043). The mediating variable, innovation capability, was measured using six items developed by Kumar and Che Rose [61]. The participants were asked to indicate their agreement level with each statement using a Likert scale consisting of five points from 1 (strongly disagree) to 5 (strongly agree). The minimum value of reliability for these items was 0.63 and the maximum value was 0.84. The CFA revealed acceptable values which are (χ2 = 19.155; Df = 15; p < 0.05; CFI = 0.963; TLI = 0.957; IFI = 0.965; RMSEA = 0.068).

The innovative climate scale encompassed five dimensions: Working Environment Support, Leader Support, Resources Supply, Learning and Growth, and Knowledge and Skills. The measurement of the innovative climate utilized a scale developed by Amabile, Conti [51], Chiou [62], and Sun, Zhao [63]. Twenty items comprised this scale, which utilized a five-point Likert scale ranging from 1 (strongly disagree) to 5 (strongly agree). The reliability of these items varied, with a minimum of 0.60 and a maximum of 0.88. After a CFA satisfactory value were obtained (χ2 = 17.158; Df = 14; p < 0.05; CFI = 0.980; TLI = 0.975; IFI = 0.988; RMSEA = 0.038). Innovative performance, as the dependent variable, was assessed using a seven-item scale that measured various performance outcomes. This scale was developed by Alpkan, Bulut [64] and Dekoulou and Trivellas [65]. Following a CFA, the acceptable values achieved (χ2 = 21.125; Df = 7; p < 0.05; CFI = 0.983; TLI = 0.976; IFI = 0.985; RMSEA = 0.075). Respondents were asked to rate their agreement with each statement on a five-point Likert scale from 1 (strongly disagree) to 5 (strongly agree). The items of the questionnaire were sourced from publicly available literature or repositories where no explicit copyright or ownership information was provided, suggesting it may be considered part of the public domain. However, the present study explicitly provided all the references for all constructs used in the questionnaire. The items exhibited a range of reliability values, the minimum being 0.75 and the maximum being 0.87, as presented in Table 4.

**Table 4:** Correlations between variables.

Variables	Items		Loading
New HRM Practices (NHRM)		(α = 0.94, AVE = 0.84, CR = 0.98)	
	NHRM1	The HR department has implemented requisite measures to mitigate the occurrence of workforce reductions.	0.70
	NHRM2	The implementation of E-recruitment makes the recruiting process in the HR department more effective.	0.62
	NHRM3	Deployment of an E-HRM system to preserve employee data and records.	0.81
	NHRM4	The HR department efficiently reassigns workers to suitable roles based on circumstances.	0.83
	NHRM5	The dedication I made to my work was duly acknowledged.	0.66
	NHRM6	Appreciating one’s role reveals how equitable the rewards system operates.	0.70
	NHRM7	The implementation of an individual performance-driven incentive mechanism.	0.61
	NHRM8	The employer gives me the freedom to make decisions about my work.	0.72
	NHRM9	When top-level decision-making is not available in the current work setting, an individual may make decisions.	0.64
	NHRM10	The Human Resources (HR) section updates staff members on company performance and business challenges.	0.75
	NHRM11	I consider myself a member of the team.	0.81
	NHRM12	Each member of the team possesses the capability to resolve the issue.	0.74
	NHRM13	Members of the team encourage creativity.	0.63
	NHRM14	Adequate work training that the company has provided for its staff.	0.67
	NHRM15	The company supports its workers in developing their skills.	0.79
	NHRM16	Learning new technologies and skills in order to compete in the market.	0.82
Innovative Climate		(α = 0.88, AVE = 0.69, CR = 0.91)	
	WES1	High autonomy and a sense of freedom are experienced throughout employment.	0.70
	WES2	Connections can provide emotional protection.	0.78
	WES3	Workers are able to foster innovation and manage non-routine issues.	0.63
	WES4	Employees’ innovative ideas and efforts were acknowledged and rewarded by the organization.	0.66
	WES5	The workplace must be easy to utilize.	0.73
	WES6	Advocate to encourage and foster innovation.	0.76
	LS7	The impression of the company leader.	0.65
	LS8	The management of unregulated subordinates.	0.68
	LS9	Facilitated the creative thought process with an open-minded approach.	0.75
	LS10	To foster an environment that is suitable to innovative work, it is imperative to establish a leadership style.	0.79
	RS11	The impression of an organization’s willingness to devote and utilize funds to foster innovation.	0.81
	RS12	When trying to facilitate the innovation capability to provide assistance in the form of financial resources, necessary materials, and relevant knowledge.	0.88
	LG13	The study examines the perception of corporate commitment to talent training and development.	0.71
	LG14	Promote employee engagement in educational endeavors.	0.78
	LG15	The goal is to enhance individuals’ abilities by fostering information sharing and exchange, hence generating additional chances for skill development.	0.69
	KS16	The enhancement of perception is done to boost the general quality.	0.60
	KS17	A perspective on the organization of skills.	0.83
	KS18	The expansion of intellectual capital.	0.80
	KS19	The aggregation of inventive endeavors.	0.64
	KS20	The implementation of novel approaches and methodologies.	0.62
Innovation Capability (NC)		(α = 0.93, AVE = 0.68, CR = 0.95)	
	NC1	The firm have the capacity to propose innovative and original concepts for new services or products.	0.73
	NC 2	The firm fosters an encouraging setting that allows individuals to create novel and valuable ideas for a range of services and products.	0.84
	NC 3	The organization believes that a generation of new and useful ideas is an important activity.	0.72
	NC 4	The firm constantly launches unique goods and services.	0.69
	NC5	The organization has developed more capabilities to apply concepts of continuous improvement and customer focus.	0.63
	NC 6	The organization has developed the ability to manage products, processes and incremental improvements and changes to the system.	0.74
Innovative Performance		(α = 0.92, AVE = 0.69, CR = 0.90)	
	IP1	The proportion of newly introduced products inside the current range of products.	0.78
	IP2	The quantity of newly initiated plans for products and services.	0.83
	IP3	The potential to offer unique goods and services to the market in advance of competition.	0.81
	IP4	Innovations are being implemented to enhance productivity and strategies.	0.84
	IP5	The assessment of the quality of recently launched products and services.	0.75
	IP6	The underlying drive behind creativity and the adaptability of employees.	0.81

### Data analysis

The analysis of all the main variables, including NHRM practices, innovative climate with its attributes, innovation capability, and innovative performance, was conducted using the statistical software Statistical Package of Social Science (SPSS) version 25.0. Hierarchical regression analysis (HRA) and PROCESS macro were employed to obtain the results. As a preconditioning step, the mean-centered values of all relevant antecedent variables were removed, and terms for interaction were subsequently generated by multiplying them with each other [66].

The present study utilized Cronbach’s Alpha to evaluate the questionnaire’s reliability. A higher value of Cronbach’s Alpha suggests greater reliability for the tested factor, indicating favorable internal uniformity of the questionnaire. The Cronbach’s Alpha value exceeded 0.8, signifying good internal uniformity. Convergent validity was assessed using the factor loading (FL) technique. Convergent validity was deemed the impact significantly because both the composite reliability (CR) and the average variance extracted (AVE) exceeded 0.5, meeting the criteria for good convergent validity (with a ratio of 0.6 being even more preferable than 0.5) [67]. The examination of the discriminant validity of the variables was undertaken by employing Confirmatory Factor Analyses (CFA) with the use of AMOS 23, to establish construct validity. The fitness findings of the model demonstrate that the data indicates a reasonable fit to the model, as presented in Table 2. Furthermore, a comparison was conducted between several measurement models (SMM) in terms of model fitness indices.

## Results

Confirmatory factor analysis (CFA) was conducted to examine the presence of common method bias. All variables in the model loading on one factor were tested. However, although common method bias was assessed using the widely accepted Harman’s one-factor test, the model fit for the data. The results show that the model consists of four common factors, and the eigenvalues of these factors are all greater than 1. Together, these factors account for 69.52 percent of the total variance. Furthermore, the investigation using the common method deviation method showed that the maximum variance associated with a single variable was 14.09 percent. The means, standard deviations, and correlations for all four variables are shown in Table 5. Hypothesis 1 sought to investigate the association between NHRM practices and innovative performance. The findings of the research indicated that NHRM practices were substantial correlated with both innovation capability (r = 0.19, p < 0.01) and innovative performance (r = 0.16, p < 0.01). Furthermore, the results of the analysis revealed a favorable correlation between innovative performance and innovation capability (r = 0.21, p < 0.01), which aligns with the initial hypotheses of this research. The findings from the HRA are obtainable in Table 6. In Model 1, it was evident that NHRM practices exhibited a significant relationship with innovative performance (ß = 0.23, p < 0.001). Thus, the results provided support for Hypothesis 1 (H1).

**Table 5:** Correlations between variables.

Sr. No	Var.	Mean	SD	1	2	3	4	5	6	7	8	9	10	11	12	13	14	15
1	Gender	0.67	0.49	1														
2	Age	2.09	0.55	0.16	1													
3	Marital Status	29.27	6.76	0.13**	0.33	1												
4	Pharmaceutical organization	0.26	0.47	0.15	0.31	0.18	1											
5	Education	3.06	0.67	0.03	-0.28	0.14	0.08	1										
6	Managerial Level	1.88	0.53	0.05	0.18	-0.13*	0.10	0.16	1									
7	Exp in Org.	4.73	4.49	0.11	0.78**	0.17	-0.17**	0.83**	0.13	1								
8	NHRM	4.58	0.64	0.05	0.15*	0.09	0.14	0.03	0.23**	0.16	1							
9	NC	4.68	0.43	0.03	0.16*	0.08	0.06	-0.03	-0.04	0.12	0.25**	1						
10	WES	4.35	0.53	0.07	0.03	0.14	0.08	0.09	0.17	0.09	0.25*	0.49**	1					
11	LS	3.68	0.35	0.12	0.10	0.09*	0.11	0.13	0.11	0.14	0.20	-0.24	0.37**	1				
12	LG	4.28	0.27	0.14	0.14	0.15	0.09	0.12	0.18	0.13	0.21	0.27	0.32*	0.35**	1			
13	RS	4.15	0.50	0.13	0.12	0.14*	0.16	0.08	0.14	0.11	0.12	0.20	0.29*	0.34	0.38**	1		
14	KS	3.77	0.24	0.09	0.11	0.13	-0.17	0.15	0.20	0.16	0.19	0.14	-0.12	0.27*	0.33	0.39**	1	
15	IP	4.76	0.71	0.06	0.16*	0.11**	0.23	0.13	0.12	0.09	0.14**	0.20**	0.16	0.16	0.14**	0.21**	0.25**	1

Note: N = 398

** p < 0.05

* p < 0.10

*** p < 0.01, NHRM (New human resource management practices), NC (Innovation capability), WES(Working Environment Support), LS(Leader Support), RS(Resources Supply), LG(Learning and Growth), KS(Knowledge and Skills), IP (Innovative performance).

The investigation into the correlation among NHRM practices and innovative performance, with the mediating factor of innovation capability, was consistent with Hypothesis H2. The investigation employed Baron and Kenny’s mediation test technique [68], which entails four essential steps. In the first and subsequent stages, it is imperative that the independent variables have a positive correlation with both the dependent variables and the mediator. During the third phase, it is expected that the mediator would demonstrate a positive association with the dependent variable. Subsequently, in the final step, the inclusion of the mediator should result in a reduction or partial attenuation of the influence of the independent variable on the dependent variable. The results of this investigation align with these steps: (1) The results of the study indicate a strong and statistically significant association between NHRM practices and innovation capability (ß = 0.34, p < 0.001); (2) innovation capability was statistically highly significant with innovative performance (ß = 0.18, p < 0.001); (3) NHRM practices exhibited a highly significant relationship with innovative performance (ß = 0.23, p < 0.001); and (4) when innovation capability was simultaneously regressed among NHRM practices and innovative performance, the effect of NHRM practices became insignificant (ß = 0.04), demonstrating a full mediation effect.

**Table 6:** Hierarchical Regression Analysis (HRA) results.

	Innovative Performance (IP)			Innovation Capability (NC)	
	M1	M2	M3	M4	M5
Gender	0.03	-0.09	0.03	-0.02	0.04
Age	-0.11	-0.07	0.09	0.02	0.01
Marital Status	0.01	0.07	0.05	0.03	0.04
Pharmaceutical Organizations	-0.04	-0.08	0.05	-0.02	0.04
Education	0.13	0.06	0.07	0.03	0.05
Managerial Level	0.10	0.09	0.04	0.05	0.01
Exp in Org.	0.07	0.13	0.06	0.02	0.05
NHRM	0.23**		0.04	0.34***	0.30***
NC		0.18***	0.23***		
WES					0.06*
LS					0.13**
LG					0.20***
RS					0.17
KS					0.09
NHRM* WES					0.17**
NHRM* LS					0.11
NHRM*RS					0.32***
NHRM*LG					0.19*
NHRM*KS					0.14*
R2	0.25	0.29	0.16	0.31	0.38
ꕔ R2	0.17	0.14	0.09	0.29	0.12
F	4.33**	5.63**	10.57**	8.45**	9.23**

**Fig 2:**
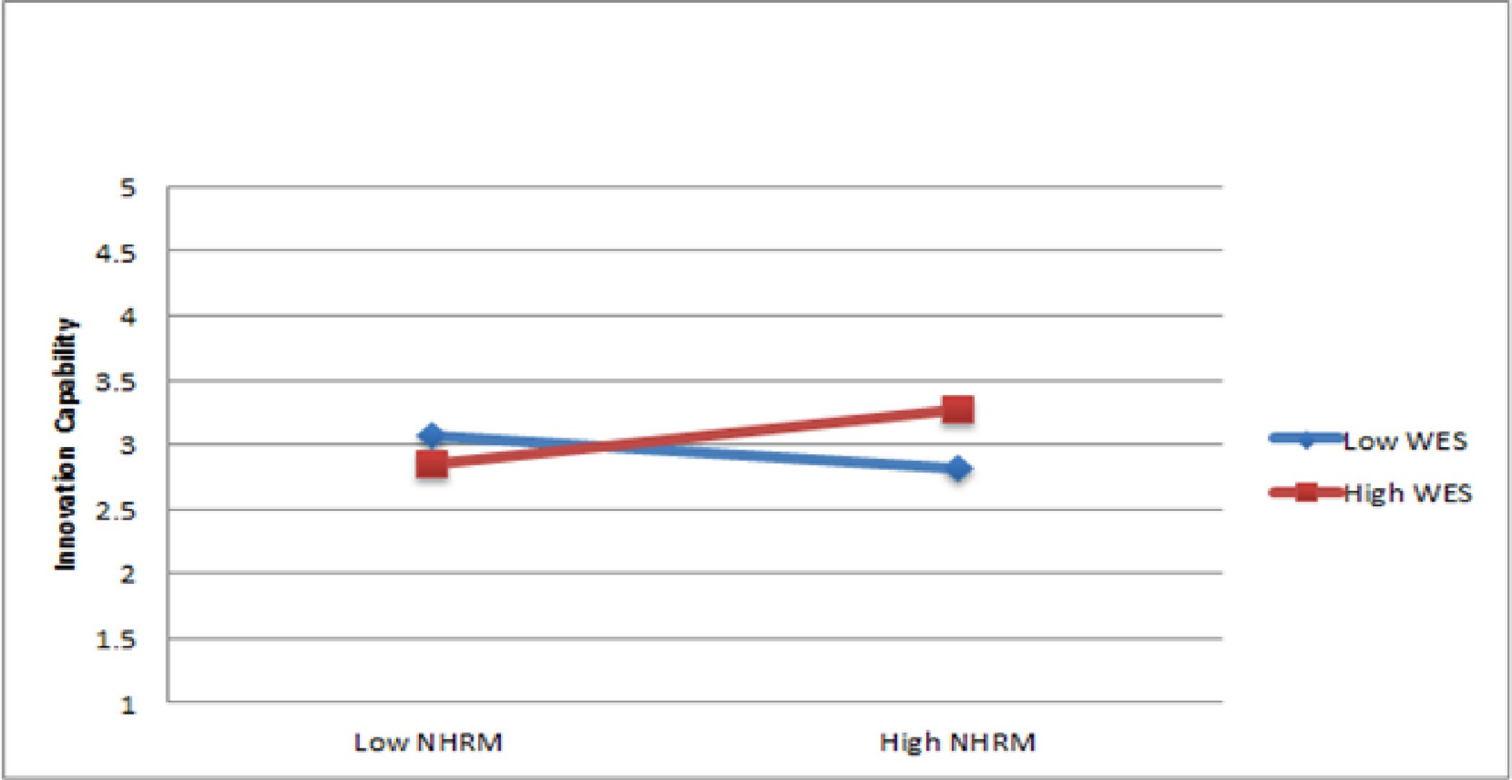
Moderating effect of work environment support.

The moderation analysis in this study followed the guidelines provided by Hayes [69], which involve utilizing the MODPROBE macro to calculate the moderating effect on complex multivariate models. The findings of Hypothesis 3a indicate that the interaction between NHRM and work environment support a strong connection to predicts innovation capability (ß = 0.17, p < 0.05). Thus, Hypothesis 3a is supported. To further illustrate the moderating impact, the significance of slopes was assessed using a test devised by Jeremy Dawson, and the slopes were graphically presented through the "Two-way interaction effect" macro in Microsoft Excel, which can be accessed at http://www.jeremydawson.co.uk/slopes.html [70]. The findings demonstrate that work environment enhances the favorable correlation between NHRM and innovation capability, as depicted in Fig 2.

**Fig 3:**
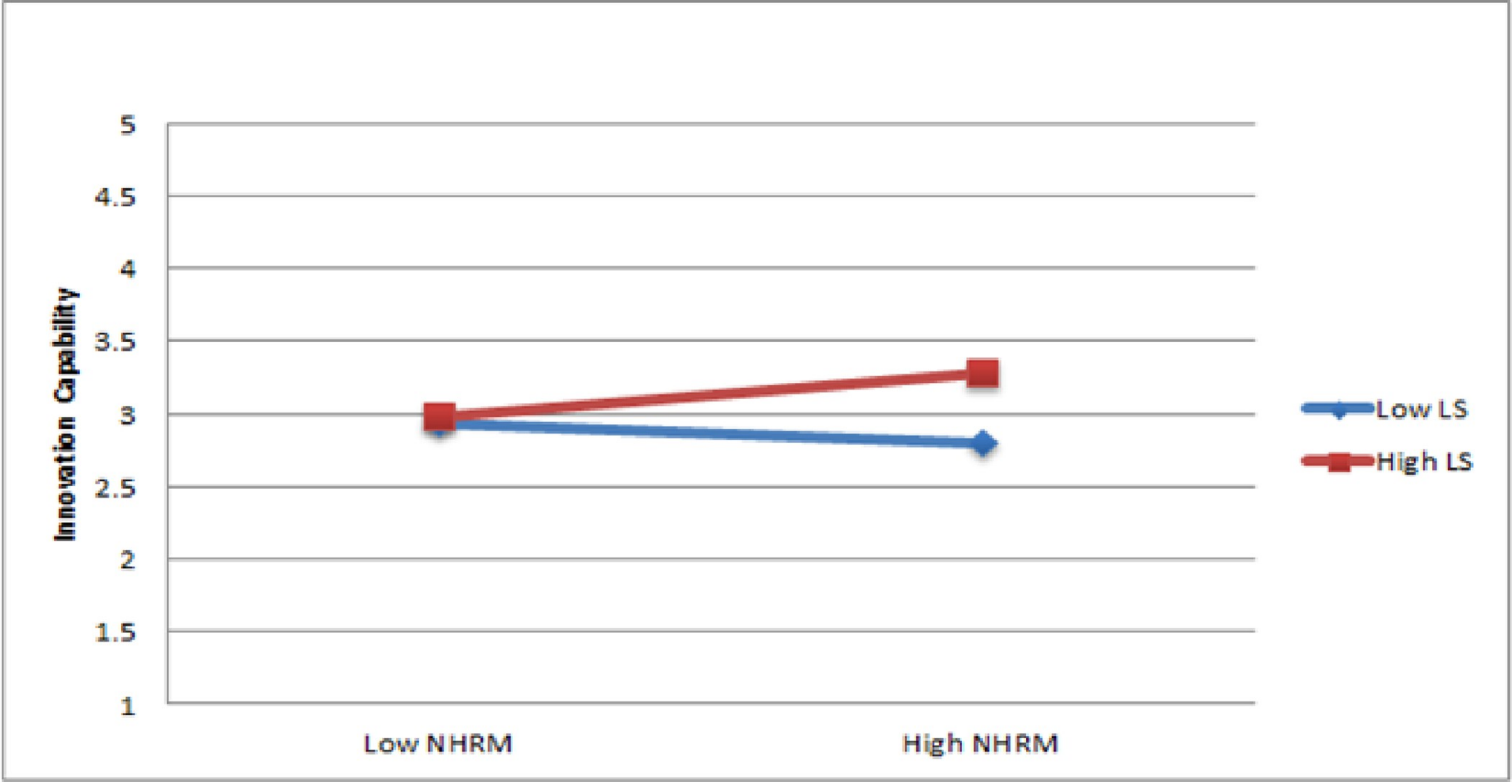
Moderating effect of leader support.

The findings of Hypothesis 3b indicate that the interaction between NHRM and leader support is not significantly predicted innovation capability (ß = 0.11, p < 0.05). Therefore, Hypothesis 3b is not supported. The presentation of the moderating impact was illustrated through a slope significance test, as shown in Fig 3. The results suggest that leader support does not strengthen the positive association between NHRM and innovation capability.

**Fig 4:**
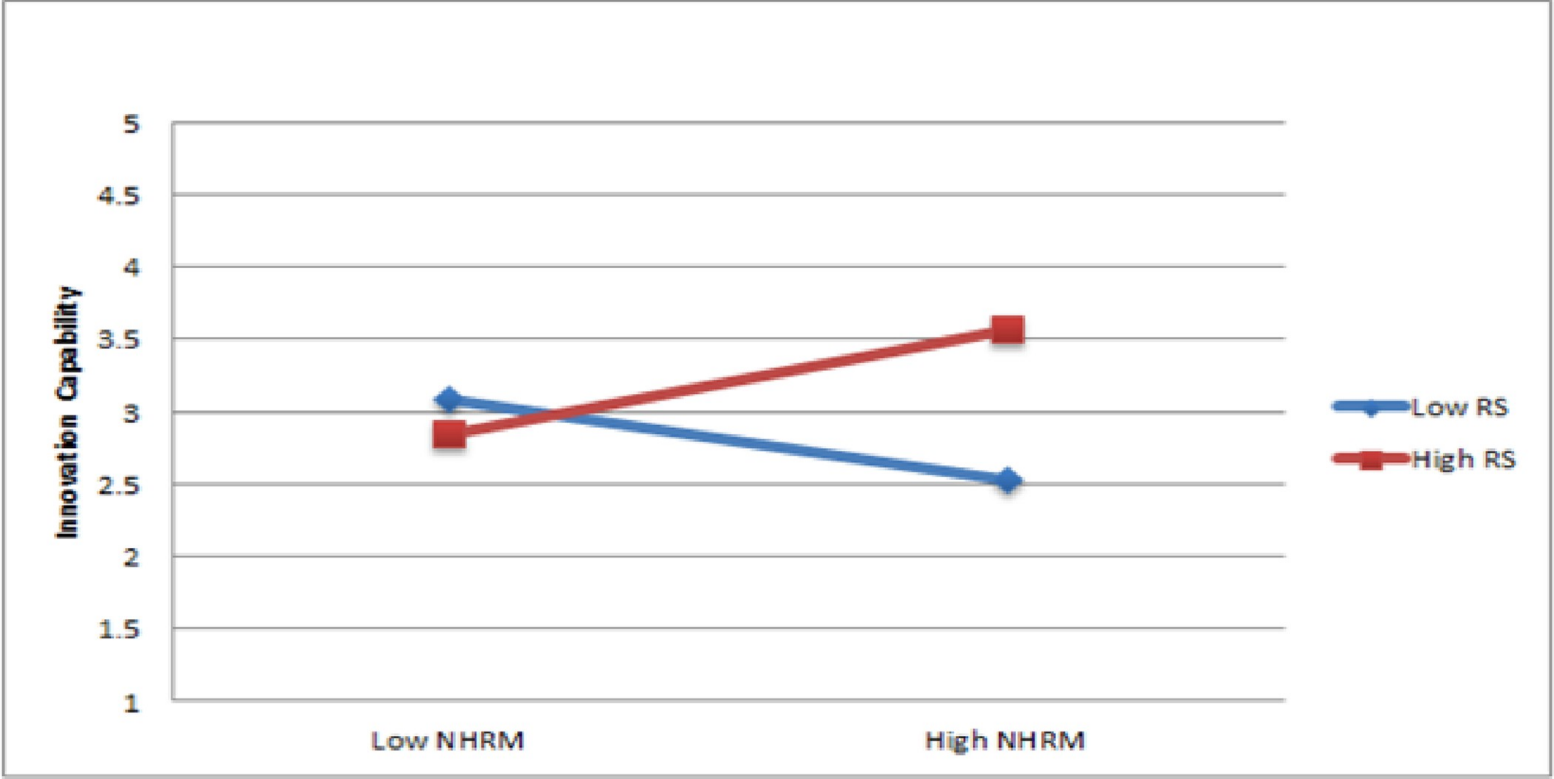
Moderating effect of resource supply.

The findings of Hypothesis 3c indicate that the interaction between NHRM and resource supply significantly estimated innovation capability (ß = 0.32, p < 0.05). Therefore, Hypothesis 3c is supported. The presentation of the moderating impact was illustrated through a slope significance test, as shown in Fig 4. The results suggest that resource supply strengthens the favorable association between NHRM and innovation capability.

**Fig 5:**
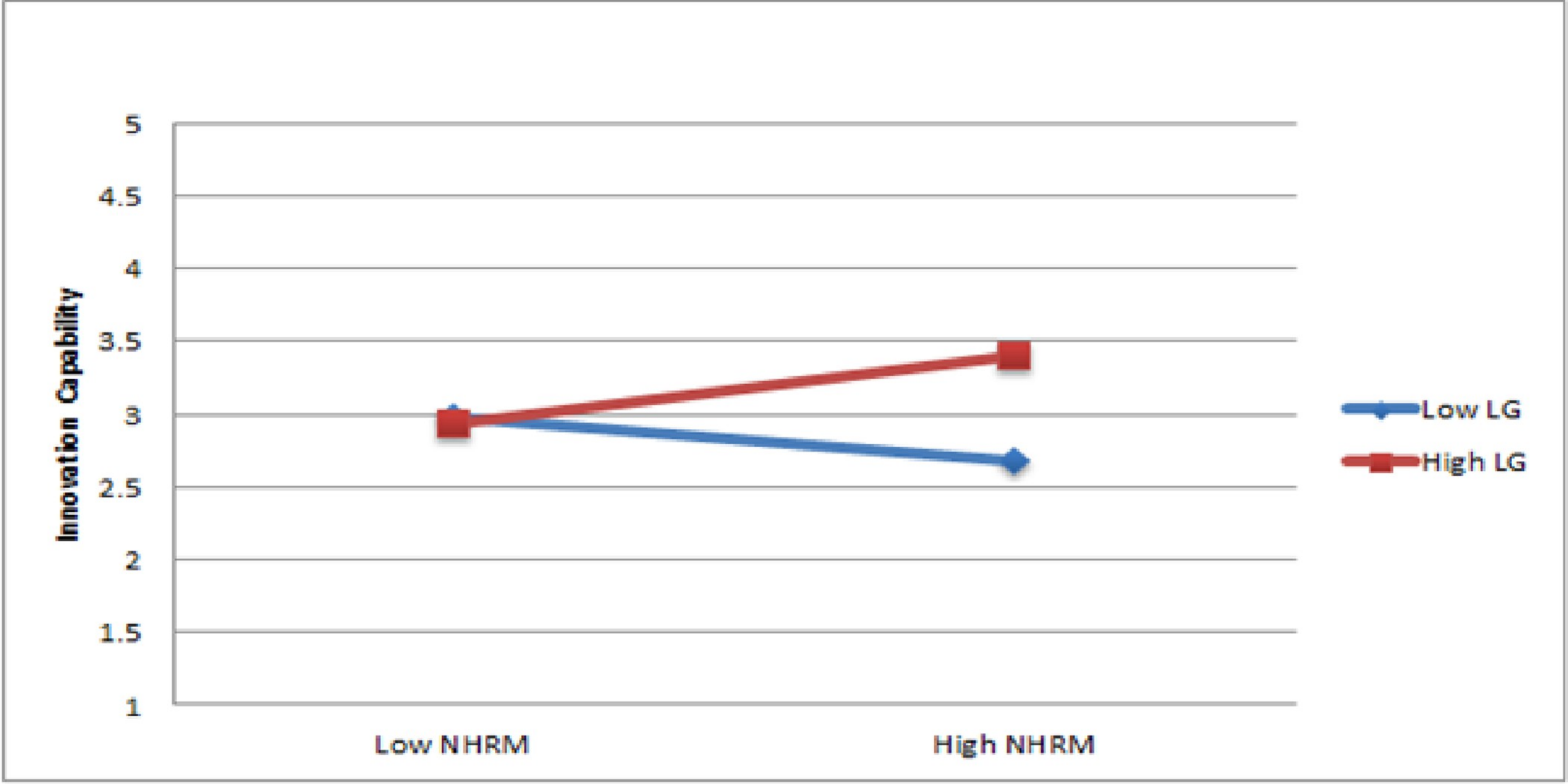
Moderating role of learning and growth.

The results of Hypothesis 3d show that the interaction between NHRM and learning and growth significantly forecasted innovation capability (ß = 0.19, p < 0.05). Therefore, Hypothesis 3d is partially supported. The presentation of the moderating impact was illustrated through a slope significance test, as shown in Fig 5. The results suggest that learning and growth strengthen the favorable connection between NHRM and innovation capability.

**Fig 6:**
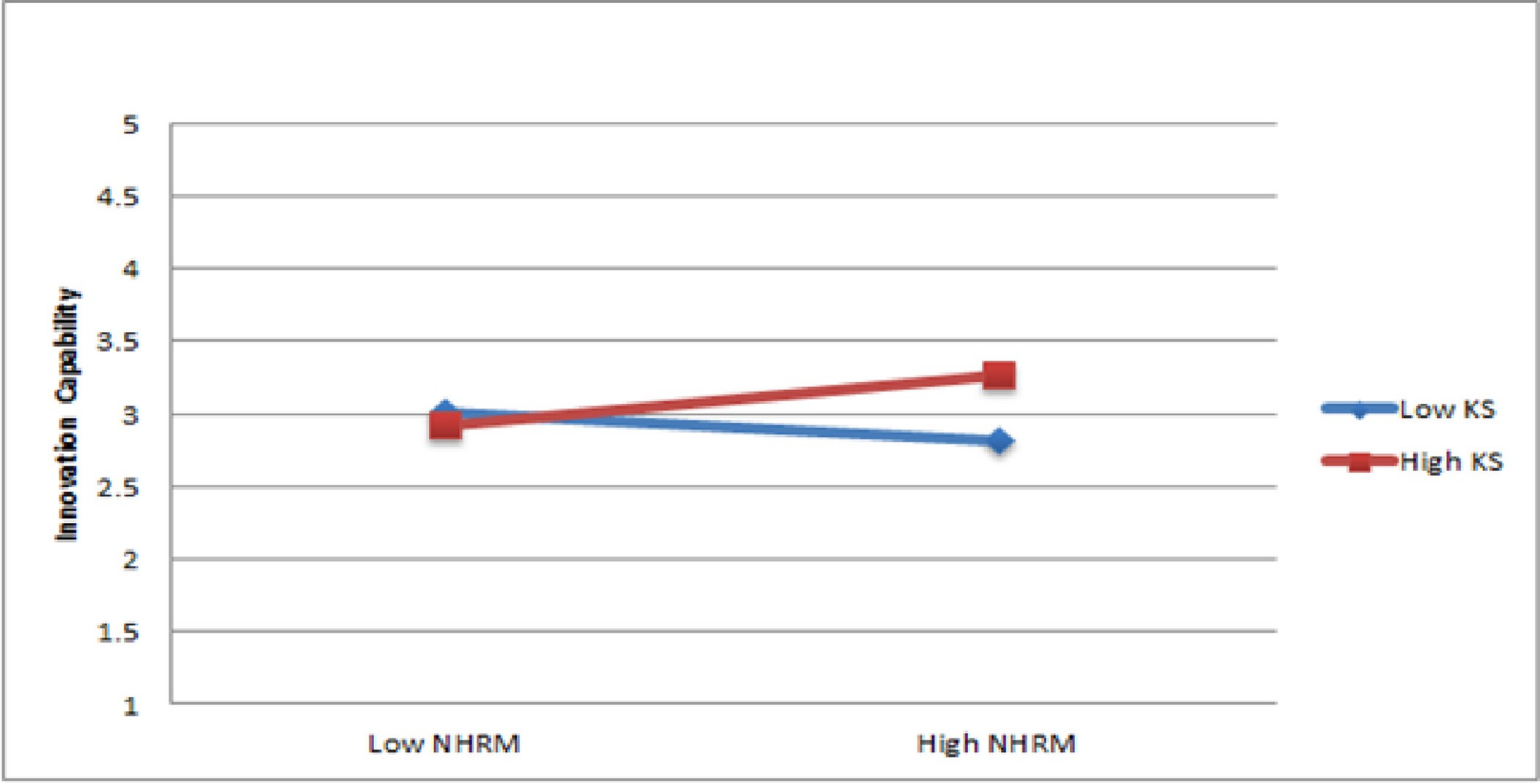
Moderating role of knowledge and skills.

The results of Hypothesis 3e indicate that the interaction between NHRM and work environment support significantly estimated innovation capability (ß = 0.14, p < 0.05). Therefore, Hypothesis 3e is partially supported. The presentation of the moderating impact was illustrated through a slope significance test, as shown in Fig 6. The results suggest that work environment support strengthens the positive link between NHRM and innovation capability.

To evaluate Hypothesis 4, which investigates the moderating mediated impact, four circumstances were evaluated as per the studies conducted by Muller, Judd [71] and Preacher, Rucker [72]. This study examines four key relationships: (1) the correlation between NHRM practices and innovative performance, (2) the connection between NHRM practices and innovative climate with its attributes, which in turn predicts innovation capability, (3) the association between innovation capability and organizational performance, and (4) the indirect impact of NHRM practices on innovative performance through innovation capability, under varying levels of innovative climate. The findings of Hypothesis 1 revealed a statistically significant favorable connection among NHRM and innovative performance, providing evidence for the initial stage (1) of moderating mediation. The findings of the moderated regression analysis are displayed in Table 6, indicating a statistically significant positive association between NHRM and innovative climate with its attributes in predicting innovation capability (ß = 0.17,0.11,0.32,0.19 and 0.14 p < 0.01, p < 0.001, p < 0.05, M5) respectively. Furthermore, Hypothesis 3 was supported (2). The results of Hypothesis 2 support Condition (3), as innovation capability has a noteworthy connection with innovative performance.

Hence, the results obtained from the three conditions demonstrate that innovative climate with its attribute moderates the mediation of innovation capability in the relationship between NHRM and innovative performance. The findings of the moderated mediation relationships further validate the study’s conclusions, following the recommendations of Preacher, Rucker [72], with the investigation conducted at both low and high degrees of innovative climate. These results presented in Table 7 indicate that findings support Hypothesis H4 and align with the four conditions outlined by Muller, Judd [71] and Preacher, Rucker [72]. H4a was also tested in accordance with these conditions, and the results further validate the study’s conclusions. It was found that the indirect effects of NHRM practices were statistically greater when a high degree of work environment support was present (IE = 0.048, SE = 0.012, p < 0.01), in contrast to the low degree work environment support condition (IE = 0.025, SE = 0.006, p < 0.01). This supports Hypothesis H4a. However, it was observed that the indirect effects of NHRM practices were not significant when a high degree of leader support was present (IE = 0.045, SE = 0.018, p < 0.01), as opposed to the low leader support condition (IE = 0.027, SE = 0.009, p < 0.01).

**Table 7:** Results of moderating mediation analysis.

Variables as Moderator	Level	Mechanism of IE	SE	95% IC LL	95% IC UL
Work Environment	High	0.048**	0.012	0.027	0.084
	Low	0.025**	0.006	0.013	0.051
	High-Low	0.019**	0.011	0.009	0.055
Leader Support	High	0.045	0.018	0.029	0.083
	Low	0.027	0.009	0.015	0.055
	High-Low	0.017	0.013	0.007	0.049
Resource Supply	High	0.047**	0.014	0.021	0.087
	Low	0.023**	0.010	0.019	0.058
	High-Low	0.025**	0.009	0.007	0.054
Learning and Growth	High	0.053**	0.013	0.025	0.081
	Low	0.030**	0.006	0.020	0.055
	High-Low	0.023**	0.011	0.004	0.047
Knowledge and skill	High	0.044	0.011	0.022	0.086
	Low	0.027	0.007	0.015	0.051
	High-Low	0.019	0.014	0.009	0.057

The results of Hypothesis H4b did not support the specified condition. It was found that the indirect effects of NHRM practices were significant in the presence of a high degree of resource supply (IE = 0.047, SE = 0.014, p < 0.01) compared to the low degree resource supply condition (IE = 0.023, SE = 0.010, p < 0.01). On the other hand, the results of Hypothesis H4c supported the condition. The findings indicated that the indirect effects of NHRM practices were significant when a high degree of learning and growth was present (IE = 0.053, SE = 0.013, p < 0.01) compared to the low degree learning and growth condition (IE = 0.030, SE = 0.006, p < 0.01). However, the findings of Hypothesis H4d did not support the condition. It was observed that the indirect effects of NHRM practices were not significant in the presence of a high degree of knowledge and skills (IE = 0.044, SE = 0.011, p < 0.01) compared to the low knowledge and skills condition (IE = 0.027, SE = 0.007, p < 0.01). Similarly, the findings of Hypothesis H4e did not support the specified condition. It was found that the indirect effects of NHRM practices were not significant in the existence of a high degree of knowledge and skills (IE = 0.044, SE = 0.011, p < 0.01) compared to the low knowledge and skills condition (IE = 0.027, SE = 0.007, p < 0.01).

## Discussion

The primary objective of this study was to fill a need in the current body of research by investigating the relationship between NHRM practices and innovative performance. To accomplish this objective, the study extensively investigated the relationship between NHRM practices and innovative performance. This investigation further examined the mediating function of innovation capacity and the moderating influence of innovative climate with its dimensions (WES, LS, RS, LG, and KS). This study has underscored the consequence of innovation capability and innovative climate as critical factors affecting innovative performance. The results of this study align with prior studies conducted by Haq, Gu [73] and Yusr, Mokhtar [74]. Therefore, the results of the current study for all hypotheses support the results of the earlier studies. The first hypothesis proposes a positive impact between NHRM practices and innovation performance related to the research results [7,24,35]. Initially, the mediation results contribute to the literature and knowledge base of human resource management. The role of innovation capability is one of the mediating effects, which is consistent with previous research, although few studies reveal the role of innovation capability as a mediating role [46]. This finding is consistent with the research of Rajapathirana and Hui [75], which shows that innovation capability positively impacts enterprises’ innovation performance. It can be seen that innovation ability is the basis for successful innovation. This finding is consistent with recent research identifying several determinants of innovation climate (WES, LS, RS, LG, and KS), emphasizing that innovation climate is a significant determinant of corporate sustainability and competitiveness [47]. In light of these results, it is evident that organizations aspiring to foster a culture of innovation and gain a competitive edge through innovation must prioritize the development of their innovation capabilities. The implementation of human resource management methods significantly influences the favorable effects on innovative performance.

This study delves into the effects of NHRM practices on innovative performance, considering the innovation capability role as mediator and the moderating role of innovative climate, including its dimensions (WES, LS, RS, LG, and KS), within the context of pharmaceutical -based organizations. Innovative climate also serves as a moderator in the association between NHRM and innovation capability. A supportive climate positively influences an innovative climate, encompassing a favorable emotional response to imposed changes and operating in a conducive environment. NHRM practices can influence various facets of performance, especially in terms of subjective evaluations. In our quest to underscore the pivotal role of NHRM practices, we emphasize their ability to improve innovative performance through the facilitation of innovative behavior, involving innovation capability and innovative climate.

### Theoretical implications

The theoretical framework of this research is grounded in NHRM practices, innovation capability, and innovative performance, with a focus on innovative climate and its attributes. This framework was empirically explored through a survey involving 398 responses from the pharmaceutical sector in Pakistan. The results have provided validation for four significant hypotheses. Firstly, the study primarily focused on investigating the impact of NHRM practices on the enhancement of creative performance in pharmaceutical companies, addressing a research gap that scholars had relatively overlooked. The study examined the significance of NHRM practices and their direct impact on innovative performance. This exploration was underpinned by the static T-O-E and RBV theory, which has gained importance as a source of advantages in competition for pharmaceutical organizations in the context of global competition. By integrating the TOE framework and RBV theory, this study can comprehensively understand how human resource management practices influence innovation performance in the pharmaceutical industry. The TOE framework provides insights into the contextual factors that shape the innovation process, while the RBV theory illuminates the role of internal resources and capabilities in driving innovation outcomes [20].

Furthermore, this study introduced the concept of innovation capability, acting as a significant intermediary within NHRM practices and innovative performance. It delved into the mediating effects related to this association. The study underscores that the effectiveness of NHRM practices is contingent upon the presence of innovative behavior. The findings emphasize the critical role of innovation capability (NC) as a fundamental resource contributing to a firm innovative performance. This observation is consistent with the research conducted by Kutieshat and Farmanesh [24] and Van Esch, Wei [55]. Such insights hold substantial theoretical relevance for researchers investigating the drivers of firm innovative performance in the context of NHRM practices. In summary, the study underscores the essential nature of innovation capability in realizing the full potential of NHRM practices and their impact on innovative performance, representing a novel development in pharmaceutical organizations.

In contrast to previous research, this study contributes a fresh perspective on the impact of NHRM practices on innovative performance. Specifically, it examines the indirect effect of NHRM practices while considering the influence of innovative climate and its associated attributes (WES, LS, RS, LG, and KS) as a moderator in the connection between NHRM practices and innovation capability. The research reveals that when the innovative climate is more favorable, the positive impact of NHRM practices on innovative performance via innovation capability becomes significantly greater. Consequently, our findings challenge the assumption of a direct association between NHRM practices and innovative performance. Instead, they highlight the intricate interplay of factors, with innovation playing a central role. In this investigation, innovation capability acts as a mediator and innovative climate with its associated attributes as a moderator forming the bedrock of the study. Moreover, this research emphasizes the significance of contextual variables, both as mediators and moderators, and underscores the essential function and efficacy of NHRM practices.

### Managerial implications

Our research findings have important ramifications for managers in various industries, but they are especially pertinent to those in the pharmaceutical sector. Pharmaceutical companies need to develop innovation capabilities because of the constant changes in regulations and technology [76], environmental disruptions like the Covid-19 pandemic, and varying expectations from stakeholders like patients, regulators, and shareholders. These skills enable people to retrieve, absorb, and integrate outside information with their knowledge. As a result, pharmaceutical managers can benefit from our results, which encourage them to spend in areas other than research and development (R&D). By leveraging their Human Resource (HR) systems, these managers can enhance innovation capabilities and improve innovative performance. Notably, New Human Resource Management (NHRM) presents a more adaptable HRM approach than control-based methods, enabling organizations to reconfigure resources effectively amidst environmental constraints.

Managers bear significant responsibility in nurturing their employees’ innovation capabilities, which, in turn, catalyze innovative performance. As such, this study carries important managerial implications aimed at enhancing innovative performance in pharmaceutical organizations, particularly in the context of Pakistan. The impact of an innovative climate, characterized by its attributes (WES, LS, RS, LG, and KS), as a moderator between NHRM practices and innovation capability (NC), underscores the importance of identifying new technologies, understanding customer preferences, and staying attuned to market trends. An innovative climate helps organizations gauge environmental dynamics and adapt by aligning with market demands. Concurrently, managers should focus on overcoming the challenges and barriers they face: resistance to change, resource constraints, leadership support, communication, and stakeholder engagement. Resistance to change can be overcome by involving employees early in the change process by communicating the rationale behind proposed recommendations and emphasizing the benefits to individuals and the organization [77]. Resource constraints can be overcome by conducting a thorough cost-benefit analysis to prioritize recommendations based on potential impact and feasibility. Look for alternative funding sources such as grants, partnerships, or budget reallocations [78]. Leadership support can be overcome by involving leaders in developing the implementation plan to ensure their involvement and commitment. It provides leadership development and coaching to equip leaders with the skills and knowledge to effectively lead change initiatives and inspire employee engagement [79]. Moreover, communication and stakeholder engagement can be overcome by developing a comprehensive communications plan outlining key message, target audiences, channels and timelines for sharing information about proposed changes. Tailor communications to different stakeholder groups, emphasizing the relevance and benefits of recommendations to their roles and interests [77]. The research provides practical implications for HRM practitioners and industry leaders in the pharmaceutical industry. Identifying the HRM practices and organizational climate dimensions contributing most to innovative performance provides actionable recommendations for improving innovation capabilities and driving organizational success. Furthermore, managers have a pivotal role in championing NHRM practices within their organizations. They should proactively foster the development of new skills, eliminate obstacles that hinder a conducive learning environment, and enhance the speed at which employees acquire and apply new skills. In doing so, NHRM practices can serve as a motivating force that empowers employees to deliver exceptional work experiences within the organization.

## Conclusion, limitations and future research directions

The current investigation has shed light on the pivotal role of NHRM practices and innovation capability in significantly enhancing and promoting innovative performance in pharmaceutical firms in Pakistan. Focusing specifically on the pharmaceutical industry, this study provides new insights into how HRM practices influence innovation, characterized by tight regulation, rapid technological advancement, and intense market competition. Furthermore, by examining this relationship in the context of Pakistan, this study addresses a significant gap in the literature, providing valuable insights relevant to academic practitioners and policymakers in the region. Therefore, this study’s contextual specificity and geographical focus add to its novelty and enrich the broader literature on human resource management, innovation and organizational performance. Due to the introduction of innovation capability as a mediator and inventive climate with its attributes as a moderator with NHRM and innovative performance factors not found in earlier studies, the study’s results are more dynamic and efficient [7,24]. While this investigation has contributed valuable information, it is essential to acknowledge its limits, which may guide research for future endeavors. First and foremost, it is important to recognize that the data in this study is by nature cross-sectional and conducted in a cross-cultural context. Future research endeavors could consider employing a longitudinal research design to access a more inclusive understanding of the dynamics involved. Additionally, qualitative methods such as in-depth interviews could be incorporated to provide deeper insights. Expanding the study’s scope to encompass diverse organizations, including private and government entities, would offer a more comprehensive perspective. Lastly, replicating the research in different countries would enable the validation of results in varying cultural and organizational contexts.

Furthermore, the exploration of its function and the impact of NHRM practices on innovative performance highlights the potential for ongoing improvement and innovation in response to evolving knowledge contexts. To ensure the accuracy and robustness of the findings, additional empirical evidence is warranted. Additionally, it would be valuable to delve into specific processes within NHRM practices, such as e-recruitment, decision-making procedures, and compensation mechanisms, to understand their impact on innovative performance more comprehensively. Another avenue for future research involves examining the moderating influence of an innovative climate on the mediator and innovation capability. While this study focused on the internal innovative climate, the influence of the external innovative climate could also be explored. Lastly, this study underscores the significant influence of NHRM practices on the innovation capability and performance of the pharmaceutical sector in Pakistan. This suggests that these organizations are not impeding the innovation process; they view innovation as a driving force for development. The results of our study highlight the need to promote a collective comprehension that aligns with the execution of HR policies and practices to develop skills and attain innovative goals. This mutual comprehension may clarify why specific organizations outperform their competitors in terms of innovation performance. This study provides significant insights into the function of NHRM in the knowledge-intensive environment and enhances the innovation of the Pakistan pharmaceutical sector.

## Supporting information

S1 File (DOCX)

S2 File (DOCX)
