# Supplementary material for: The HR revolution: Redefining performance paradigms in Pakistan’s pharma landscape through moderating role of innovative climate
Source: PLoS One. 2024 May 31;19(5):e0301777. doi: 10.1371/journal.pone.0301777 (PMC11142687; doi:10.1371/journal.pone.0301777)
Supplement: S1 File — (DOCX) [file pone.0301777.s001.docx]

**Informed Consent Form**

Title of Research Project: The HR Revolution: Redefining Performance Paradigms in Pakistan’s Pharma Landscape Through Moderating Role of Innovative Climate

Principal Investigator: Abdul Waheed & Shahbaz Hussain

Affiliation: Jiangsu University & University of Okara

Thank you for your interest in helping us understand how new HRM practices can benefit organizations! This form provides information about our research study and asks for your consent to participate. Please read carefully and feel free to ask any questions before deciding.

This study has been approved by the Central Academic Review Board (CARB) under reference number 743. We aim to investigate how the new HRM practices and innovation capability by an innovative climate can contribute to improved innovative performance in Pharma Industry. By understanding these connections, we can develop practical recommendations and strategies for companies to become more innovative.

By participating, you'll answer a few questions about your company's new HRM practices, innovation capability, innovative climate, and innovative performance. It should take only 10-15 minutes of your time. Remember, your participation is completely voluntary and confidential. Your name and other identifying information will never be disclosed in any reports or publications.

Your contribution will have a real impact. Your insights will help us develop valuable research on new HRM practices, innovation capability, innovative climate, and innovative performance. You'll also receive updates on our findings, so you can see how your participation made a difference.

Together, we can make a lasting impact on the planet. Thank you for considering your participation!

If you have any questions about the study, please contact: [**raihussain@uo.edu.pk**](mailto:raihussain@uo.edu.pk).

Consent:

I have read and understand the information provided above. I freely and voluntarily consent to participate in this study.

Name and Signature:

Date:
