# Supplementary material for: The HR revolution: Redefining performance paradigms in Pakistan’s pharma landscape through moderating role of innovative climate
Source: PLoS One. 2024 May 31;19(5):e0301777. doi: 10.1371/journal.pone.0301777 (PMC11142687; doi:10.1371/journal.pone.0301777)
Supplement: S2 File — (DOCX) [file pone.0301777.s002.docx]

**QUESTIONNAIRE**

The HR Revolution: Redefining Performance Paradigms in Pakistan’s Pharma Landscape Through Moderating Role of Innovative Climate

**Dear Sir/Madam,**

In order to better understand performance paradigms in Pakistan, we kindly ask for your cooperation in completing this survey. Your feedback is greatly appreciated and will help us improve the new HRM practices and innovation capability by an innovative climate.

This is a research project being conducted by the research group at the University of Okara. Your participation in this survey investigation is voluntary. Your answers will be confidential, and only be assessed by the research group for research purposes. It will take about 05 minutes to complete the survey. Please provide answers to all questions as it is important to research applications.

If you have any questions about the study, please contact: [**raihussain@uo.edu.pk**](mailto:raihussain@uo.edu.pk)

According to your experience in the Pharma Industry, please indicate to which extent you agree on the following statements.

**Demographic Information**

**Gender**: (1) Male (2) Female

**Marital status**: (1) Single (2) Married

**Age:** (1) 20-30 (2) 31-40 (3) 41-50 (4) Over 50 years

**Please indicate your managerial level:** (1) Coordinator (2) Supervisor (3) Senior Manager

**Education:**  (1) Technical Degree (2) Bachelor (3) Post Graduate (4) MPHIL (5) PhD

**Experience in the pharmaceutical industry**: (1) ≤ One Year (2) 1-5 Years (3) 6-10 Years (4) 11-15 years (5) ≥ 15 Years

**Research Variables Information**

Dear Respondent! Kindly select the level on your judgment based on five points Likert scale ranging from (1=Strongly Disagree to 5=Strongly Agree)

| **New HRM Practices (NHRM)** | Strongly Disagree | Disagree | Neither agree nor Disagree | Agree | Strongly Agree |
| --- | --- | --- | --- | --- | --- |
| The HR department has implemented requisite measures to mitigate the occurrence of workforce reductions. | 1 | 2 | 3 | 4 | 5 |
| The implementation of E-recruitment makes the recruiting process in the HR department more effective. | 1 | 2 | 3 | 4 | 5 |
| Deployment of an E-HRM system to preserve employee data and records. | 1 | 2 | 3 | 4 | 5 |
| The HR department efficiently reassigns workers to suitable roles based on circumstances. | 1 | 2 | 3 | 4 | 5 |
| The dedication I made to my work was duly acknowledged. | 1 | 2 | 3 | 4 | 5 |
| Appreciating one's role reveals how equitable the rewards system operates. | 1 | 2 | 3 | 4 | 5 |
| The implementation of an individual performance-driven incentive mechanism. | 1 | 2 | 3 | 4 | 5 |
| The employer gives me the freedom to make decisions about my work. | 1 | 2 | 3 | 4 | 5 |
| When top-level decision-making is not available in the current work setting, an individual may make decisions. | 1 | 2 | 3 | 4 | 5 |
| The Human Resources (HR) section updates staff members on company performance and business challenges. | 1 | 2 | 3 | 4 | 5 |
| I consider myself a member of the team. | 1 | 2 | 3 | 4 | 5 |
| Each member of the team possesses the capability to resolve the issue. | 1 | 2 | 3 | 4 | 5 |
| Members of the team encourage creativity. | 1 | 2 | 3 | 4 | 5 |
| Adequate work training that the company has provided for its staff. | 1 | 2 | 3 | 4 | 5 |
| The company supports its workers in developing their skills. | 1 | 2 | 3 | 4 | 5 |
| Learning new technologies and skills in order to compete in the market. | 1 | 2 | 3 | 4 | 5 |
| **Innovative Climate (IC)** | | | | | |
| High autonomy and a sense of freedom are experienced throughout employment. | 1 | 2 | 3 | 4 | 5 |
| Connections can provide emotional protection. | 1 | 2 | 3 | 4 | 5 |
| Workers are able to foster innovation and manage non-routine issues. | 1 | 2 | 3 | 4 | 5 |
| Employees' innovative ideas and efforts were acknowledged and rewarded by the organization. | 1 | 2 | 3 | 4 | 5 |
| Connections can provide emotional protection. | 1 | 2 | 3 | 4 | 5 |
| The workplace must be easy to utilize. | 1 | 2 | 3 | 4 | 5 |
| Advocate to encourage and foster innovation. | 1 | 2 | 3 | 4 | 5 |
| The impression of the company leader. | 1 | 2 | 3 | 4 | 5 |
| The management of unregulated subordinates. | 1 | 2 | 3 | 4 | 5 |
| Facilitated the creative thought process with an open-minded approach. | 1 | 2 | 3 | 4 | 5 |
| To foster an environment that is suitable to innovative work, it is imperative to establish a leadership style. | 1 | 2 | 3 | 4 | 5 |
| The impression of an organization's willingness to devote and utilize funds to foster innovation. | 1 | 2 | 3 | 4 | 5 |
| When trying to facilitate the innovation capability to provide assistance in the form of financial resources, necessary materials, and relevant knowledge. | 1 | 2 | 3 | 4 | 5 |
| The study examines the perception of corporate commitment to talent training and development. | 1 | 2 | 3 | 4 | 5 |
| Promote employee engagement in educational endeavors. | 1 | 2 | 3 | 4 | 5 |
| The goal is to enhance individuals' abilities by fostering information sharing and exchange, hence generating additional chances for skill development. | 1 | 2 | 3 | 4 | 5 |
| The enhancement of perception is done to boost the general quality. | 1 | 2 | 3 | 4 | 5 |
| A perspective on the organization of skills. | 1 | 2 | 3 | 4 | 5 |
| The expansion of intellectual capital. | 1 | 2 | 3 | 4 | 5 |
| The aggregation of inventive endeavors. | 1 | 2 | 3 | 4 | 5 |
| The implementation of novel approaches and methodologies. | | | | | |
| **Innovation Capability (NC)** | | | | | |
| The firm have the capacity to propose innovative and original concepts for new services or products. | 1 | 2 | 3 | 4 | 5 |
| The firm fosters an encouraging setting that allows individuals to create novel and valuable ideas for a range of services and products. | 1 | 2 | 3 | 4 | 5 |
| The organization believes that a generation of new and useful ideas is an important activity. | 1 | 2 | 3 | 4 | 5 |
| The firm constantly launches unique goods and services. | 1 | 2 | 3 | 4 | 5 |
| The organization has developed more capabilities to apply concepts of continuous improvement and customer focus. | 1 | 2 | 3 | 4 | 5 |
| The organization has developed the ability to manage products, processes and incremental improvements and changes to the system. | 1 | 2 | 3 | 4 | 5 |
| **Innovative Performance (IP)** | | | | | |
| The proportion of newly introduced products inside the current range of products. | 1 | 2 | 3 | 4 | 5 |
| The quantity of newly initiated plans for products and services. | 1 | 2 | 3 | 4 | 5 |
| The potential to offer unique goods and services to the market in advance of competition. | 1 | 2 | 3 | 4 | 5 |
| Innovations are being implemented to enhance productivity and strategies. | 1 | 2 | 3 | 4 | 5 |
| The assessment of the quality of recently launched products and services. | 1 | 2 | 3 | 4 | 5 |
| The underlying drive behind creativity and the adaptability of employees. | 1 | 2 | 3 | 4 | 5 |

***Thank you for Your Participation.***
